# Multimodal imaging of bilateral ischemic retinal vasculopathy associated with Berger’s IgA nephropathy: case report

**DOI:** 10.1186/s12886-021-01935-1

**Published:** 2021-05-08

**Authors:** Khaled El Matri, Francesca Amoroso, Olivia Zambrowski, Alexandra Miere, Eric H. Souied

**Affiliations:** 1 Department of Ophthalmology, Centre Hospitalier Intercommunal de Créteil, University Paris Est Créteil, 40 Avenue de Verdun, 94000 Créteil, France; 2 Department B, Institut Hedi Rais d’ophtalmologie de Tunis, University Tunis – El Manar, Boulevard 9 Avril 1938, 1006 Tunis, Tunisia

**Keywords:** Multimodal imaging, OCT-Angiography, Retinal vasculopathy, Berger IgA nephropathy, Case report

## Abstract

**Background:**

Berger’s IgA nephropathy (IgAN) is the most common primary glomerulonephritis. However, some rare cases of retinal manifestations have been described, with only two cases of retinal vasculopathy reported in the literature.

Here we report an uncommon case of bilateral ischemic retinal vasculopathy associated with Berger IgAN, evaluated with complete multimodal imaging including ultra-wide field (UWF) imaging and swept source optical coherence tomography angiography (SS-OCTA).

**Case presentation:**

A 51-year-old woman with a history of Berger’s IgA nephropathy complained of visual impairment in both eyes. Fundus examination showed bilateral peripapillary arterial attenuation and perivascular sheathing, associated to perifoveal telangiectatic lesions. There was a central scotoma in the perimetry of the right eye and peripheral visual field defect in the left eye. Full-field electroretinogram revealed significantly reduced oscillatory potentials. Spectral domain optical coherence tomography showed multiple focal areas of thinning of the inner retina, indicating long-lasting vascular occlusion lesions. UWF fluorescein angiography showed the presence of bilateral vasculitis, diffuse capillary leakage, macular ischemia and telangiectasia. SS-OCTA better highlighted the macular ischemia and vascular anomalies layer-by-layer.

**Conclusions:**

Retinal vasculopathy is a very rare condition observed in IgA nephropathy. To our knowledge, this is the first report of complete multimodal functional and structural imaging. UWF imaging was very useful for accurate and comprehensive disease assessment, and OCTA was able to assess posterior pole vascular lesions.

## Background

IgA nephropathy (IgAN), also known as Berger’s disease, is the most common primary glomerulonephritis, usually presenting with hematuria that occurs during an upper respiratory infection, associated with mild proteinuria [1, 2]. IgAN occurs mainly in children and young adults and has a wide range of clinical associations with various inflammatory diseases [3–5]. The hallmark of the disease is the widespread deposition of IgA antibodies in the mesangium of the glomerula and activation of complement cascade with mesangial and endocapillary hypercellularity [5–7].

Ocular manifestations associated with IgAN range from scleritis and episcleritis [8, 9] to keratoconjunctivitis and anterior uveitis [10, 11]. A few rare cases of retinal manifestations have been described [12, 13], with only two cases of retinal vasculopathy reported in the literature [14].

We report a case of a patient presenting with bilateral ischemic retinal vasculopathy associated with Berger IgAN. Functional and structural abnormalities of the retina were assessed thanks to full-field electroretinogram (ff-ERG), perimetry and complete multimodal imaging including ultra-wide field (UWF) imaging and swept-source optical coherence tomography angiography (SS-OCTA).

## Case presentation

A 51-year-old Caucasian woman presented to the University of Creteil Eye Hospital complaining of lack of improvement in visual acuity two months after cataract surgery in both eyes. The patient had a medical history of auto-immune cholangiopathy, Berger’s IgA nephropathy and a family history of an unspecified eye tumor in her father. No consanguinity was reported in the family. Berger’s IgAN has been diagnosed 24 years ago, following pregnancy and it has been previously treated with corticosteroids. Patient is under angiotensin receptor blockers (Irbesartan) successfully controlling her blood pressure for the last 24 years.

Auto-immune cholangiopathy was diagnosed 10 years ago and it is currently under control, treated with ursodeoxycholic acid (Delursan).

Best-corrected visual acuity was 20/63 on the right eye (RE) and 20/40 on the left eye (LE). Slit-lamp examination did not show any sign of anterior segment inflammation, pupillary light reflex was preserved and intraocular pressure was normal in both eyes.

**Fig. 1:**
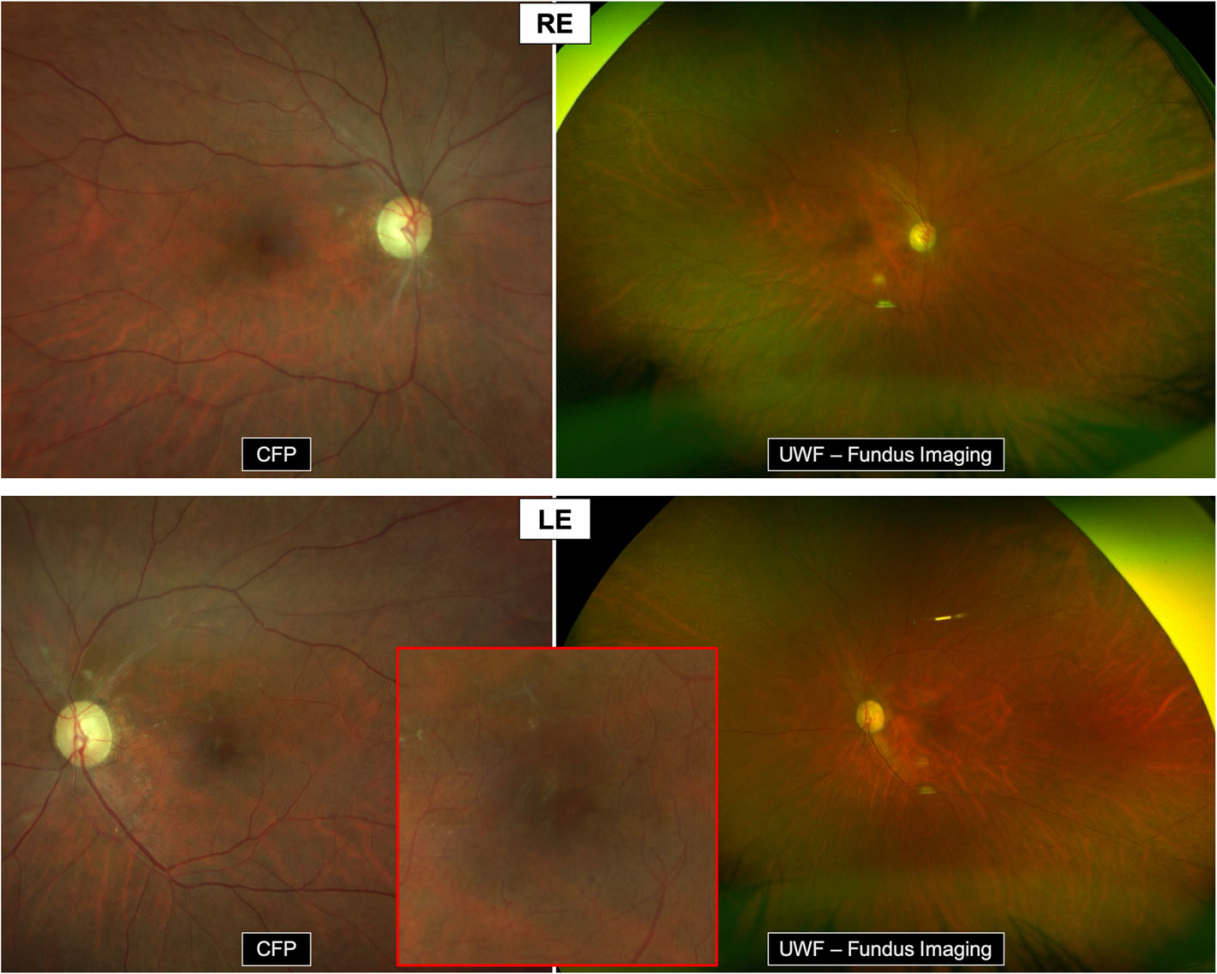
Fundus imaging. CFP: Normal appearance of the fovea and the optic nerve head. Bilateral sectorial vascular attenuation and perivascular sheathing of the peripapillary retinal arterioles. Bilateral multiple telangiectatic lesions around the fovea. Red square = magnification of the macula in LE enhancing the perifoveal capillary telangiectasia. No retinal haemorrhages. UWF – Fundus imaging: No peripheral lesions

Fundus examination and colour fundus photography (CFP) (*Clarus 500, Zeiss, Germany*) revealed a preserved foveal reflex and a normal aspect of the optic nerve head. However, sectorial vascular attenuation and perivascular sheathing affecting the peripapillary retinal arterioles has been noted in both eyes. Bilateral multiple telangiectatic lesions were present around the fovea, without retinal hemorrhage or any other associated lesion. UWF fundus imaging (*Optomap, Optos California, USA)* did not reveal any peripheral lesion (Fig. 1).

**Fig. 2:**
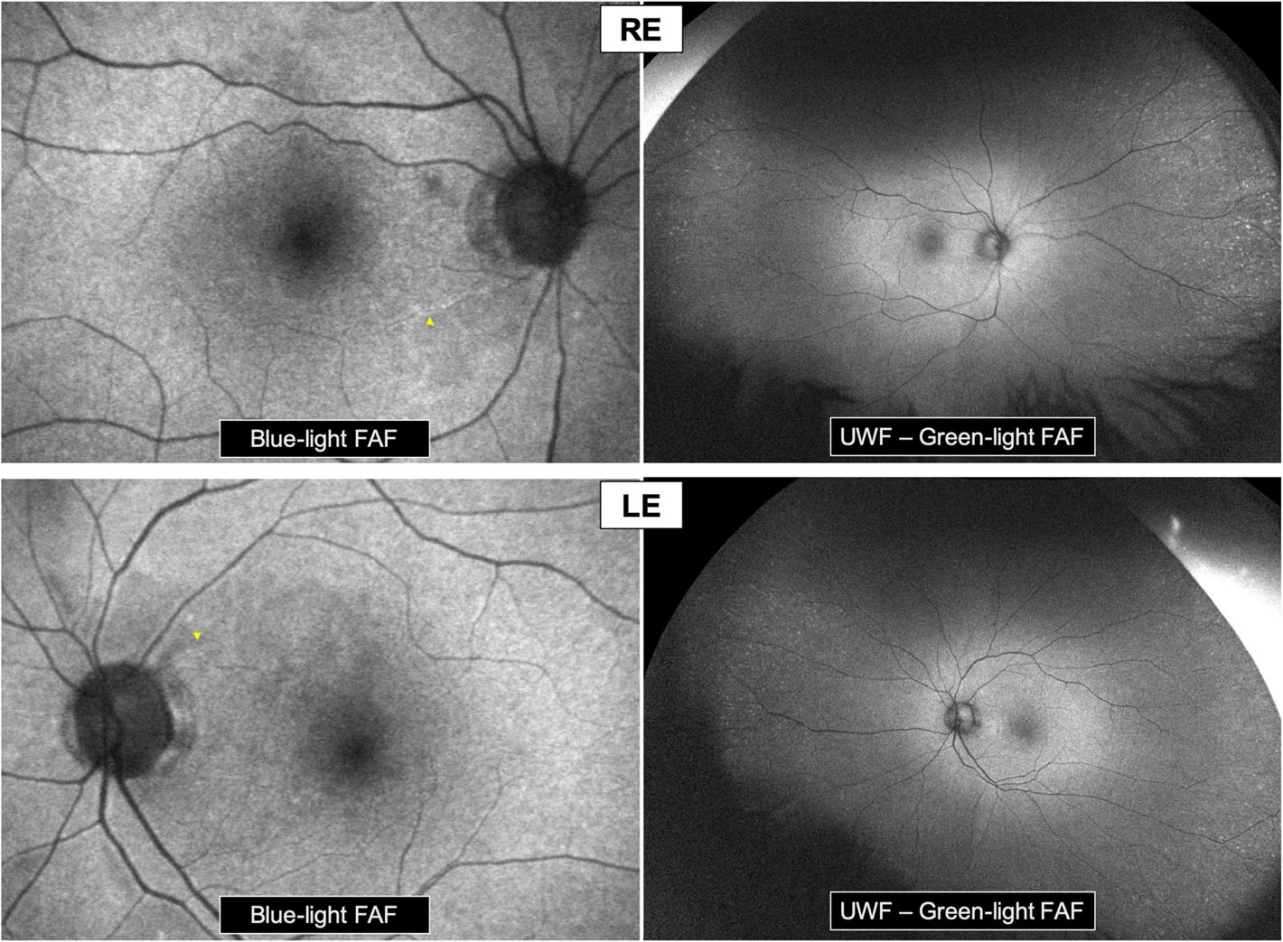
Fundus autofluorescence. Blue-light FAF of the posterior pole: Heterogenous autofluorescence of the perimacular area. Flair attenuation of some peripapillary arterioles (yellow arrowheads). UWF – Green-light FAF: Normal autofluorescence of the mid-periphery. Presence of hyper-autofluorescent spots in the extreme periphery in both eyes

Blue-light fundus autofluorescence (FAF) *(Spectralis-HRA2, Heidelberg, Germany)* of the posterior pole showed heterogenous autofluorescence of the perimacular area and a flair attenuation of some peripapillary arterioles. UWF green-light FAF (*Optomap, Optos California, USA)* highlighted the presence of hyper-autofluorescent spots in the extreme periphery, while the mid-periphery was normal (Fig. 2).

**Fig. 3:**
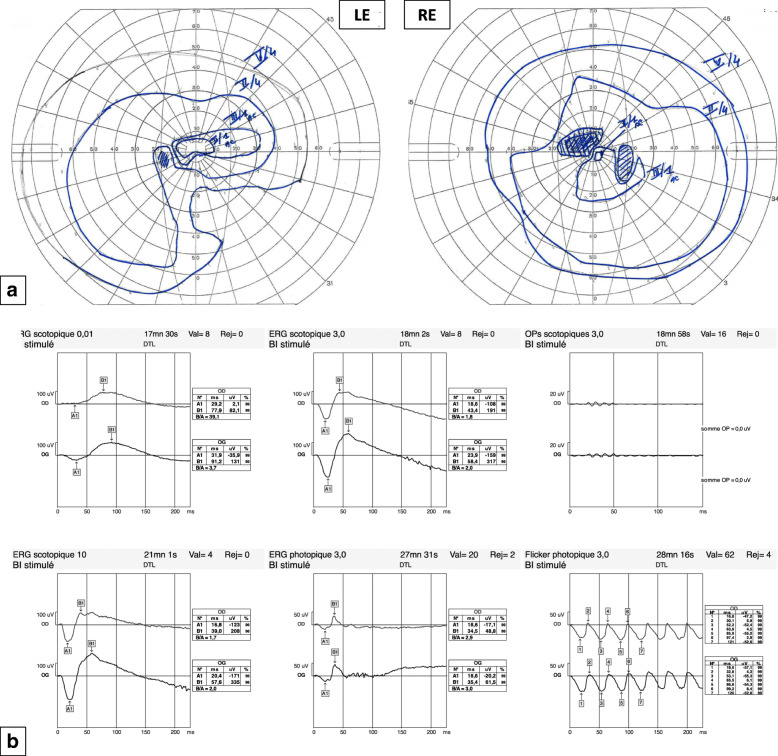
Functional campimetry and electrophysiology assessment. **a**- GVF perimetry: RE: Absolute superior paracentral scotoma. Enlarged blind spot. LE: Sectorial inferior nasal defect. **b**- Full field ERG: Normal appearance of scotopic and photopic responses tracings. Amplitude asymmetry with diminished amplitude in RE. Significant decrease in oscillatory potentials in both eyes (barely discernible from background noise)

Goldmann visual field (GVF) *(MT-325UD, Takagi, Japan)* showed an absolute superior paracentral scotoma with an enlarged blind spot in the RE and a sectorial inferior nasal defect in the LE (Fig. 3a).

On the full-field electroretinogram (ff-ERG) *(Metrovision, France)*, a significant decrease in oscillatory potentials in both eyes, which were barely discernible from the background noise, was identifiable. Otherwise, the scotopic and photopic ERG responses were normal in appearance with amplitude asymmetry, being diminished in the RE (Fig. 3b).

**Fig. 4:**
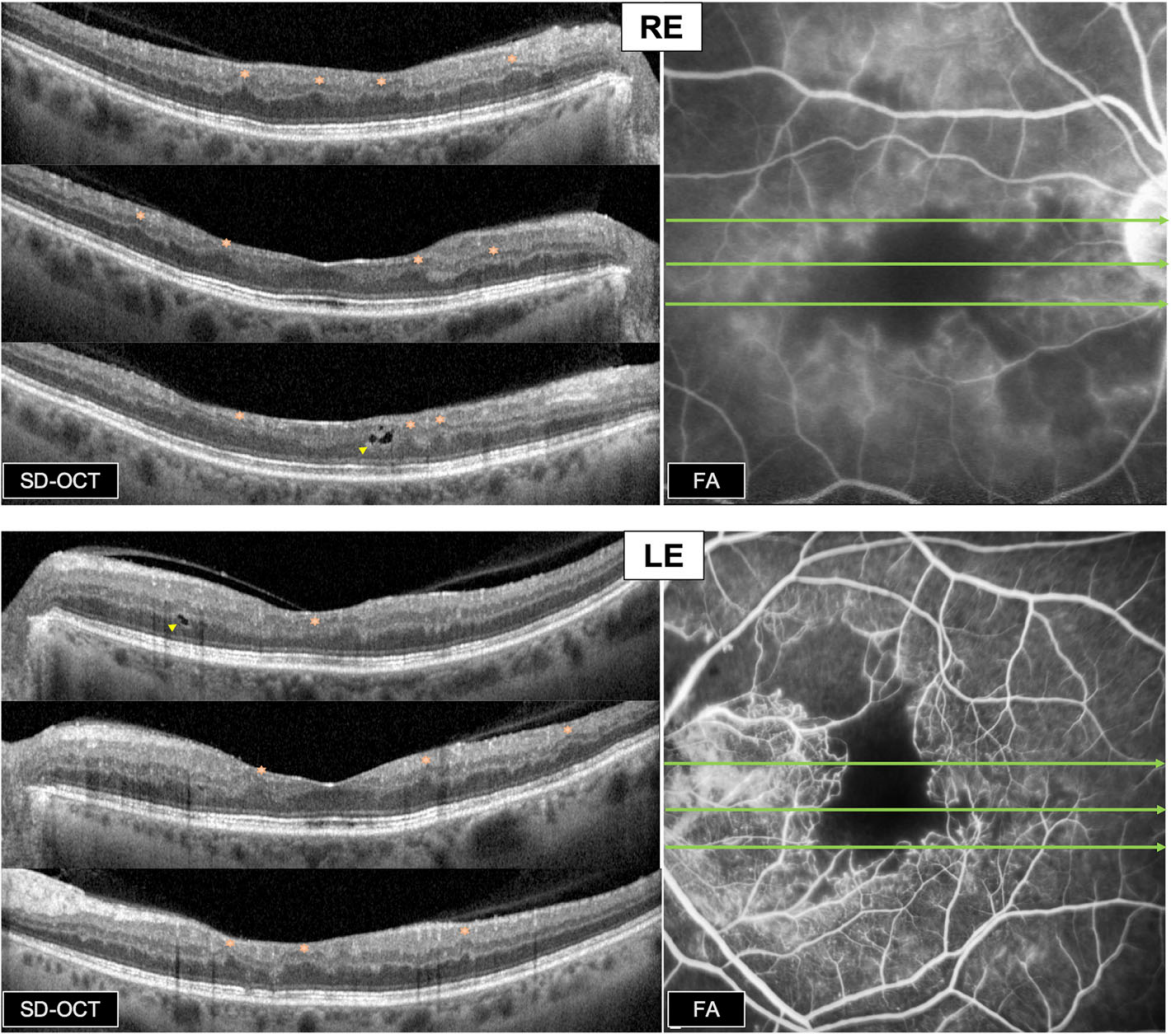
Structural SD-OCT macular scans and corresponding macular FA images. SD-OCT: Multiple focal areas of inner retinal thinning (GCL, INL & OPL thinning), associated to corresponding ONL relative thickening, in both eyes (orange hexagrams). Presence of intraretinal hyporeflective cavities in both eyes (yellow arrowheads). FA: Bilateral macular ischemia, capillary occlusions and telangiectasia. RE: Central ischemia with significant enlargement of FAZ. LE: Arched appearance of the ischemic lesion. Green arrows = The different levels of SD-OCT scans

Spectral domain optical coherence tomography (SD-OCT) *(Spectralis-HRA2, Heidelberg, Germany)* passing through the macula showed multiple focal areas of ganglion cell layer (GCL), inner nuclear layer (INL) and outer plexiform layer (OPL) thinning, associated to corresponding outer nuclear layer (ONL) relative thickening, in both eyes; the presence of intraretinal hypo-reflective degenerative cavities was also highlighted (Fig. 4).

**Fig. 5:**
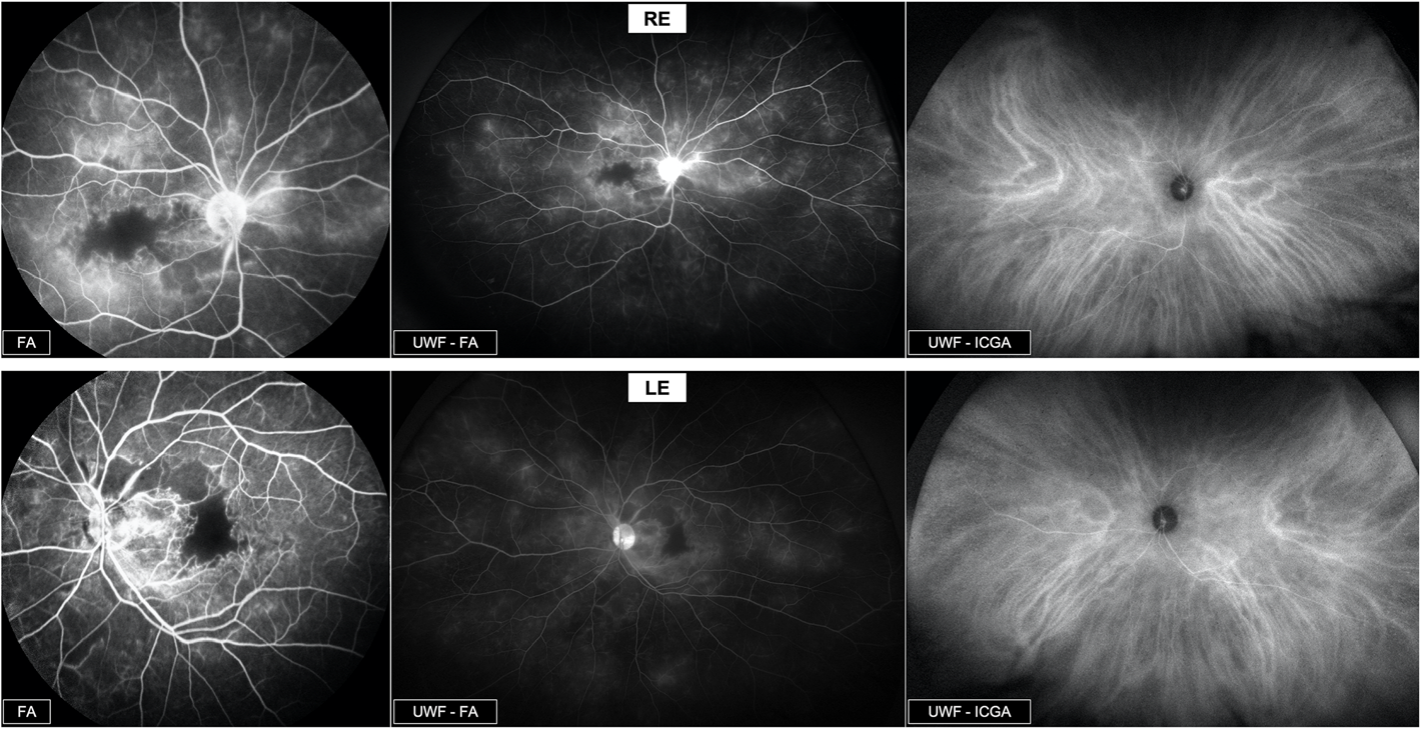
Fluorescein and ICG angiography. FA & UWF - FA: Bilateral retinal vasculitis with multiple focal areas of perivascular dye leakage and diffuse capillary leakage extending from the posterior pole to the periphery. Bilateral macular ischemia with capillary occlusions and telangiectasia. UWF – ICGA: No particular lesion in mid-phase ICGA

Fluorescein angiography (FA) *(Spectralis-HRA2, Heidelberg, Germany / Optomap, Optos California, USA)* showed bilateral retinal vasculitis with multiple focal areas of perivascular dye leakage and diffuse capillary leakage extending from the posterior pole to the periphery. FA showed the presence of bilateral macular ischemia with capillary occlusions and telangiectasia. The macular lesions were mainly central in the RE, with significant enlargement of the foveal avascular zone (FAZ), while ischemic lesion had an arched appearance in the LE. (Figs. 4 and 5).

Infracyanine green angiography (ICGA) *(Spectralis-HRA2, Heidelberg, Germany / Optomap, Optos California, USA)* was unremarkable in both eyes (Fig. 5).

### Besides, circulatory filling times were normal in both FA and ICGA

**Fig. 6:**
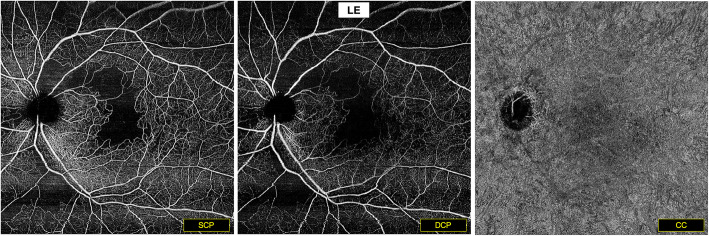
En face SS-OCT-A (12x12 mm) of the LE. Well-delineated superior arched macular ischemia. Capillary drop-out visible in both SCP and DCP, associated to shunt vessels and telangiectatic capillaries at the borders of the ischemic area. Nasal inferior area of capillary drop-out in the SCP and DCP slabs. No particular lesion at the level of the CC slab

En face SS-OCT-A 12 × 12 mm *(PlexElite, Zeiss, Germany)* showed the superior arched area of macular ischemia detected on FA in the LE, with a well-delineated arched capillary drop-out in both superficial capillary plexus (SCP) and deep capillary plexus (DCP). Shunt vessels and telangiectatic capillaries were observed at the borders of the ischemic area. A nasal inferior area of capillary drop-out was also noted in the SCP and DCP slabs. Outer retinal, choriocapillaris (CC) and choroidal slabs analysis were unremarkable. SS-OCTA acquisition in the RE was laborious due to poor fixation of the patient, with low resolution barely interpretable images (Fig. 6).

Blood pressure measured during the visit was within the normal range. The patient was referred to her nephrologist for a general evaluation to eventually initiate immunosuppressive therapy.

According to her physician, blood pressure was under control and her blood work was unremarkable. The patient was put on oral corticosteroids (Prednisolone) 0.5 mg/kg per day (30 mg daily), followed by progressive reduction in corticosteroids doses. At her last control, 4 months following initial assessment, patient was on Prednisolone 2.5 mg daily. BCVA improved to 20/40 in RE following laser capsulotomy and was maintained 20/40 in LE. However, retinal vasculitis was still active on FA, especially on the LE. The patient was referred back to her physician to eventually put her back on high doses of corticosteroids.

## Discussion and conclusions

Here we describe a rare case of bilateral retinal vasculopathy with diffuse vasculitis complicated with bilateral macular ischemia in a patient with history of auto-immune diseases associating auto-immune cholangitis and Berger’s IgA nephropathy.

The patient was assessed with comprehensive multimodal imaging, including functional (GVF, ff-ERG, and FAF) and structural (SD-OCT, FA, ICGA, and SS-OCT-A) evaluation.

Berger’s disease is the most common primary glomerulonephritis [2]. The presence of circulating IgA immune complexes and their deposition in extrarenal vessels indicates systemic involvement of IgAN [6, 7]. Ocular involvement in patients with IgAN is rare [15], however, the most frequent association occurs with scleritis and episcleritis including one case of bilateral posterior scleritis, followed by keratoconjunctivitis and anterior uveitis [8–11, 16, 17]. Rare cases of ciliochoroidal effusion, and subretinal drusenoid deposits have also been reported [12, 13, 18].

Wolfensberger et al. [14] described for the first time the association of Berger’s IgAN and retinal vasculopathy in the two cases reported so far. The first patient described by Wolfensberger et al. [14] complained of visual loss in her RE secondary to a slight vitreous hemorrhage. Fundus examination and imaging showed the presence of bilateral vascular anomalies such as venous dilations, telangiectasia, capillary occlusions and peripheral retinal vascular proliferation. Berger’s IgAN diagnosis was made after ocular manifestations, on renal biopsy that was requested because of anamnesis of recurrent hematuria and epistaxis. The second patient described by the same authors complained of bilateral visual loss secondary to mild vasculitis with bilateral venous occlusion associated to central macular edema, parallel to an acute episode of hematoma in a context of renal failure. Diagnosis was made on immunoelectrophoresis showing increased levels of IgA.

In our case, the patient complained of a lack of improvement in visual function after cataract surgery in both eyes. Initial examination revealed vascular attenuation, with presence of visual field defects on GVF perimetry. Ff- ERG was performed in the presence of a history of auto-immune diseases to rule-out auto-immune retinopathy, showing asymmetric scotopic and photopic responses within normal limits associated with a significant decrease in oscillatory potentials in both eyes, reflecting dysfunction of amacrine cells and indicating a macular lesion.

Drug-induced retinal toxicity was ruled-out as well, since the patient has been treated with angiotensin receptor blockers (Irbesartan) controlling her blood pressure, and ursodeoxycholic acid (Delursan) for the auto-immune cholangiopathy and neither treatment has been previously reported as toxic for the retina or retinal vessels. Besides, patient has been previously treated with corticosteroids for her nephropathy, which probably accelerated the onset of her cataract.

FA showed the presence of bilateral vasculitis with diffuse capillary leakage, and bilateral macular ischemia with large areas of capillary drop-out, explaining the functional symptoms. The appearance of the macular/perimacular non-perfusion territories corresponded to the visual field defects and scotomas on GVF. Furthermore, the presence of multiple focal areas of GCL, INL, and OPL thinning and paradoxical apparent ONL thickening on SD-OCT is consistent with chronic paracentral acute middle maculopathy lesions [19], reflecting long standing arteriolar occlusions. Besides, intraretinal hypo-reflective cavities noted on SD-OCT are also consistent with the chronic nature of the aforementioned lesions. Hypertensive retinopathy is unlikely responsible for these chronic occlusive lesions, since the patient is treated with angiotensin receptor blockers, successfully controlling her blood pressure for the last 24 years.

SS-OCT-A precisely delimited the areas of perimacular capillary drop-out in the LE, layer-by-layer, with high resolution images and highlighted microvascular changes as shunt vessels and telangiectasia, without dye leakage disturbance as in classic angiography. However, poor fixation due to significant macular ischemia in the RE made SS-OCT-A acquisition laborious with low quality images.

The pathogenesis of retinal vascular anomalies in Berger’s IgAN is still unknown. Wolfensberger et al. [14] hypothesized that deposition of IgA immune complexes in retinal vessels and capillaries, similar to the deposits observed at the level of the glomerula, would induce localized vascular changes with local inflammation, leading to platelets adhesion, aggregation and thrombosis with retinal ischemia. These vascular and structural changes could also be caused by lysosomal enzymes release from polynuclear leukocytes, ultimately increasing perivascular tissues damage.

Our patient also had a history of autoimmune cholangitis and some rare cases of uveitis have been reported in association with autoimmune liver diseases [20], including anterior and intermediate uveitis but no cases of retinal vasculitis or vascular abnormalities had been reported before. Therefore, the chronic retinal vasculopathy observed in our patient is more likely to be related to Berger’s IgAN.

Retinal vasculopathy is a very rare condition observed in IgA nephropathy. Anamnesis of hematuria or presence of renal function deterioration should make physicians consider the diagnosis. Multimodal retinal imaging and especially ultra-wide field imaging are very useful for accurate and comprehensive lesion evaluation. Additionally, SS-OCT-A can non-invasively assess posterior pole vascular lesions with high resolution layer-by-layer analysis.

## Data Availability

All data supporting our findings are contained within the manuscript.

## Abbreviations

IgAN: IgA nephropathy
UWF: Ultra-wide field
SS-OCTA: Swept source optical coherence tomography angiography
ff-ERG: Full-field electroretinogram
RE: Right eye
LE: Left eye
FAF: Fundus autofluorescence
GVF: Goldmann visual field
SD-OCT: Spectral domain optical coherence tomography
GCL: Ganglion cell layer
INL: Inner nuclear layer
OPL: Outer plexiform layer
ONL: Outer nuclear layer
FA: Fluorescein angiography
FAZ: Foveal avascular zone
ICGA: Infracyanine green angiography
SCP: Superficial capillary plexus
DCP: Deep capillary plexus
CC: Choriocapillaris
